# Oxidative Stress Biomarkers and Senescence-Related Gene Expression in Human Lumbar Degenerative Disc Disease

**DOI:** 10.3390/molecules31213792

**Published:** 2026-10-22

**Authors:** Charles A. Carazzo, Alexandre P. Tognon, Fernando A. C. Xavier, Luciana B. A. Touguinha, Catia S. Branco, Daniel R. Marinowic, Manuela Peletti-Figueiró, Asdrubal Falavigna

**Affiliations:** 1 Health Sciences Postgraduate Program, University of Caxias do Sul (UCS), Caxias do Sul 95070-560, RS, Brazil; 2 Cell Therapy Laboratory, University of Caxias do Sul (UCS), Caxias do Sul 95070-560, RS, Brazil; 3 São Vicente de Paulo Hospital, Passo Fundo 99010-080, RS, Brazil; aptognon@hsvp.com.br; 4 Brain Institute of Rio Grande do Sul (BraIns), Pontifical Catholic University of Rio Grande do Sul (PUCRS), Porto Alegre 90619-900, RS, Brazil; 5 Medicine and Health Sciences Graduate Program, Medical School, Pontifical Catholic University of Rio Grande do Sul (PUCRS), Porto Alegre 90619-900, RS, Brazil; 6 Oxidative Stress Laboratory, University of Caxias do Sul (UCS), Caxias do Sul 95070-560, RS, Brazilcsbranc1@ucs.br (C.S.B.)

**Keywords:** lumbar degenerative disc disease, oxidative stress, cellular senescence, gene expression, biomarkers, sulfhydryl proteins, nitric oxide

## Abstract

**Background:** Oxidative stress is increasingly recognized as a key driver of lumbar degenerative disc disease (LDDD), promoting inflammation, extracellular matrix degradation, and cellular senescence. However, studies integrating oxidative stress biomarkers with senescence-related gene expression in human intervertebral discs remain limited. **Methods:** Thirty-two patients undergoing lumbar spine surgery were prospectively enrolled, yielding 36 surgically obtained intervertebral disc specimens. Thirty patients with symptomatic LDDD comprised the study population, while two patients with Pfirrmann grade I discs served exclusively as laboratory controls. Specimens were classified as mild (Pfirrmann grades II–III) or severe degeneration (grades IV–V). Due to tissue availability, RT-qPCR analyses were performed on 34 specimens, whereas oxidative stress biomarkers were evaluated in 32 specimens. Oxidative stress was assessed by measuring sulfhydryl protein levels and nitric oxide production, while the expression of NF-κB, GSK3β, and P21 was quantified by RT-qPCR. **Results:** Severely degenerated discs showed significantly lower sulfhydryl protein levels than mildly degenerated discs (*p* = 0.009), indicating impaired antioxidant defense. P21 ΔCq values were significantly higher in severe degeneration, indicating lower relative P21 expression (*p* = 0.038), whereas NF-κB and GSK3β showed no significant differences between degeneration groups. Significant positive correlations among NF-κB, GSK3β, and P21 suggested coordinated variation in these molecular pathways. Nitric oxide levels tended to be higher in severe degeneration but were not significantly different. **Conclusions:** Advanced LDDD was associated with impaired antioxidant capacity and lower relative P21 expression. Reduced sulfhydryl protein levels and altered P21 expression support an association between oxidative stress, senescence-related pathways, and disc degeneration.

## 1. Introduction

Lumbar degenerative disc disease (LDDD) is one of the leading causes of chronic low back pain and disability worldwide, representing a substantial public health and socioeconomic burden. According to the Global Burden of Disease Study 2021, low back pain affects approximately 619 million people worldwide, and this number is projected to increase to 843 million by 2050 [1,2]. LDDD is currently recognized as a multifactorial disorder resulting from the interaction of aging, genetic susceptibility, mechanical loading, and biochemical alterations that progressively impair intervertebral disc homeostasis [3,4,5].

Recent evidence has identified oxidative stress as a key biological mechanism involved in disc degeneration. Reactive oxygen species (ROS), which are continuously generated during normal cellular metabolism, become harmful when their production exceeds the antioxidant capacity of the tissue. This imbalance promotes mitochondrial dysfunction, oxidative DNA damage, extracellular matrix degradation, and activation of inflammatory signaling pathways, ultimately accelerating intervertebral disc degeneration [6,7,8,9,10,11]. Rather than representing a simple disturbance of the oxidant–antioxidant balance, oxidative stress is currently understood as a disruption of redox signaling and regulation, resulting in widespread alterations in cellular homeostasis and stress-response pathways [12].

The antioxidant defense system is essential for maintaining redox homeostasis and limiting oxidative damage. It comprises both enzymatic antioxidants, such as superoxide dismutase and catalase, and non-enzymatic antioxidant molecules, including glutathione and sulfhydryl-containing compounds [13,14]. Progressive impairment of these defense mechanisms contributes to persistent oxidative stress, promoting degradation of extracellular matrix components and amplification of inflammatory signaling. In particular, ROS-mediated activation of the NF-κB pathway enhances the production of pro-inflammatory cytokines and matrix-degrading enzymes, establishing a self-perpetuating cycle that accelerates tissue degeneration [15,16,17].

Oxidative stress has also emerged as an important trigger of cellular senescence. Persistent oxidative DNA damage activates cell-cycle regulatory pathways, particularly the p53–P21 axis, leading to irreversible growth arrest and the development of the senescence-associated secretory phenotype (SASP). This phenotype is characterized by sustained secretion of inflammatory mediators and catabolic factors that further compromise disc homeostasis. Previous studies have demonstrated increased expression of senescence-related markers, including p53, P21, and Rb, in degenerated human intervertebral discs, supporting the concept that cellular senescence plays a central role in disease progression [18,19].

Although experimental models have substantially advanced the understanding of oxidative stress during disc degeneration [20], studies simultaneously evaluating biochemical oxidative stress biomarkers together with senescence-related gene expression in surgically obtained human intervertebral disc tissue remain scarce. Such an integrated approach may provide a more comprehensive characterization of the molecular mechanisms underlying LDDD and facilitate the identification of biologically relevant biomarkers.

Therefore, the present study aimed to investigate oxidative stress in human lumbar degenerative disc disease by combining biochemical assessment of sulfhydryl protein levels and nitric oxide production with the evaluation of senescence-related gene expression (NF-κB, GSK3β, and P21) using RT-qPCR. We hypothesized that advanced disc degeneration would be associated with impaired antioxidant defense and altered expression of genes related to inflammation and cellular senescence.

## 2. Methodology

### 2.1. Ethics Statement

The study was conducted in accordance with the Declaration of Helsinki (2013) and approved by the Ethics Committee of the University of Caxias do Sul, Brazil (CEP/UCS 2.503.156; CAAE 2668520.0.0000.5341). Written informed consent was obtained from all participants prior to enrollment.

### 2.2. Patient Selection

Patients with symptomatic lumbar degenerative disc disease (LDDD) who failed conservative treatment and subsequently underwent lumbar surgery at our institution were prospectively recruited. Conservative management included analgesics, anti-inflammatory medication, postural education, and physical therapy. Surgical treatment was indicated based on persistent symptoms associated with radiological findings and clinical judgment. Patients with previous lumbar surgery, spinal infection, or neoplastic disease were excluded.

### 2.3. Radiological and Clinical Evaluation

Lumbar magnetic resonance imaging (MRI) was performed using T2-weighted fast spin-echo sequences. Disc degeneration was graded according to the Pfirrmann classification (grades I–V) [21]. For statistical analysis, specimens were categorized as mild degeneration (grades II–III) or severe degeneration (grades IV–V), as previously described [22].

### 2.4. Samples

Thirty-two patients undergoing surgical treatment for lumbar degenerative disc disease were prospectively enrolled in this study. Thirty patients with symptomatic lumbar degenerative disc disease comprised the study population included in the clinical and statistical analyses (Table 1), whereas two patients with Pfirrmann grade I intervertebral discs were included exclusively as laboratory controls and were not included in the clinical or statistical analyses. A total of 36 surgically obtained intervertebral disc specimens were collected, with four patients contributing specimens from two lumbar levels. Each specimen was independently classified according to the Pfirrmann grading system before laboratory processing.

Because tissue availability varied after surgical collection and sample processing, not all specimens were available for every experimental analysis. RT-qPCR analyses were performed on 34 specimens (6 mild and 28 severe degeneration), whereas oxidative stress biomarkers were evaluated in 32 specimens (16 mild and 16 severe degeneration).

For all laboratory analyses, the intervertebral disc specimen was considered the experimental unit because each specimen represented an anatomically distinct intervertebral disc with its own radiological degeneration grade and independent molecular and biochemical characterization. Clinical characteristics are summarized in Table 1.

### 2.5. Obtaining and Isolating the Intervertebral Disc

The IVLD fragments excised during the surgical procedure underwent a dual phase cleaning process within sterile containers containing a physiological solution to eliminate potential contamination of blood cells. Subsequently, the material was immersed in: I- sterile alkaline phosphate buffer (comprising PBS 1× with 0.8% NaCl, 0.02% KH_2_PO_4_ and 0.088% Na_2_HPO_4_, with a pH of 7.4—sourced from LGC Biotecnologia in São Paulo, Brazil) and were then transported to the Cell Therapy Laboratory. In the Laboratory, the collected material was evaluated and measurements, ensuring the standardization of biological samples approximately sized at 2 cm × 1 cm, each containing a 1:1 ratio of annulus fibrosus (AF) and nucleus pulposus (NP). These standardized samples were then immersed in 10% buffered formalin at a pH of 7.4 for a fixation period. Verification of specific intervertebral lumbar disc (IVLD) regions was carried out by a neurosurgeon and clinical researcher and corroborated through histological evaluation using hematoxylin and eosin (H&E) staining for the accuracy of samples to be evaluated by molecular biology and oxidative stress studies. This paraffin-embedded material was utilized for RT-qPCR. For ROS evaluation, samples were incubated in RNAlater (Sigma-Aldrich^®^, St. Louis, MO, USA), following the same selection criteria for parts of the IVLD and stored in an ultrafreezer until the beginning of the analyses.

### 2.6. Deparaffinization and RNA Extraction for RT-qPCR

Approximately 0.7 g of tissue (containing 1:1 proportions of IVLD structures) was used for RT-qPCR analyses. The paraffin block containing the tissue was cut using a Leica RM 2120 RT microtome (Wetzlar, Germany) and the sections were transferred to microtubes with the addition of 300 µL of mineral oil. The samples were vortexed and incubated at 90 °C for 20 min. Tissue samples were prepared for extraction using the MagMAX^TM^ Viral/Pathogen Nucleic Acid Isolation Kit (ThermoFisher, Waltham, MA, USA) and processed in the KingFisher^TM^ Duo Prime automated extractor (ThermoFisher, Waltham, MA, USA) for RNA extraction.

### 2.7. Quantification of Gene Expression by RT-qPCR

RNA samples were quantified using spectrophotometry (260 nm/280 nm) with NanoDrop^TM^ (ThermoFisher, Waltham, MA, USA). Subsequently, the RNA was converted to cDNA using the GoScript^TM^ Reverse Transcriptase kit (Sigma-Aldrich^®^, St. Louis, MO, USA), following the manufacturer’s instructions. This reaction was performed in the Veriti^TM^ thermal cycler (ThermoFisher, Waltham, MA, USA). The cDNA samples were stored at −20 °C until quantification of RT-qPCR. Each sample was diluted to the same concentration (150 ng/µL). For the reaction, PowerUp^TM^ SYBR^TM^ Green Master Mix (ThermoFisher, Waltham, MA, USA) was used, with 10 µL of master mix, 50 pmol of each pair of oligonucleotides, and 2 µL of the diluted samples. Oligonucleotides for the ACTB gene (β-actin) were used as the endogenous reference for normalization of the target genes. Gene expression was analyzed using ΔCq values, calculated as the difference between the Cq of each target gene and the Cq of ACTB (ΔCq = Cq_target − Cq_ACTB). ΔCq values were used for the statistical comparisons between mild and severe degeneration groups. The genes and oligonucleotide sequences utilized are those outlined in Table 2.

### 2.8. Cell Isolation and Lysis

Cell isolation from the samples for biochemical analyzes of oxidative stress was performed on an average total of 0.7 g of tissue obtained surgically (1:1 AF and NP). The material was dissociated enzymatically using 0.2 mg/mL of Pronase (Sigma-Aldrich^®^, St. Louis, MI, USA) for 1 h and 1 mg/mL of collagenase type IA (Sigma-Aldrich^®^, St. Louis, MI, USA) for 2 h in a 37 °C water bath, followed by successive agitation. Subsequently, the cell suspension (pool of obtained cells—fibroblasts and chondrocytes) was centrifuged and resuspended in 300 µL of Lysis buffer (Tris HCl 0.1 M pH 8.0; EDTA 0.5 M pH 8.0; and SDS 0.2%) supplemented with 5 µL of Proteinase K (5 mg/mL, Sigma-Aldrich^®^, St. Louis, MI, USA). The samples were then incubated in a 37 °C water bath for 2 h with agitation. After centrifugation, the cellular supernatant was stored at −80 °C until the start of the analyses [23,24].

### 2.9. Total Protein Quantification

The total protein content was determined using bovine serum albumin as standard, read at 595 nm on a spectrophotometer (Victor-X3, Perkin Elmer, Turku, Finland) and its result was utilized in subsequent tests [25].

### 2.10. Production of Nitric Oxide (NO)

Griess reagent was prepared with 1% sulfanilamide added to a 0.1% solution of N-1-naphthyl ethylenediamine dihydrochloride in a 1:1 (*v*/*v*) ratio. Subsequently, 50 μL of cellular supernatant was added to 96-well plates, followed by the addition of 50 μL of Griess reagent. The reaction was then incubated for 15 min in the dark. The readings were taken at 540 nm using a spectrophotometer (Victor-X3, Perkin Elmer, Turku, Finland). The results were expressed in nanomoles of nitrite per gram of protein and the evaluation was carried out in triplicate [26].

### 2.11. Quantification of Sulfhydryl Protein

The sulfhydryl protein was determined through a reaction with 5,5′-dithiobis (2-nitrobenzoic acid—DTNB—Sigma-Aldrich^®^, St. Louis, MI, USA), read at 260 nm on a spectrophotometer (Victor-X3, Perkin Elmer, Turku, Finland). The results were expressed in nanomoles of DTNB per gram of protein and the evaluation was carried out in triplicate [27].

### 2.12. Statistical Analysis

Statistical analyses were performed using IBM SPSS Statistics version 27 (IBM Corp., Armonk, NY, USA). Continuous variables are presented as mean ± standard deviation (SD), whereas categorical variables are presented as absolute frequencies. Comparisons between mild and severe degeneration groups were performed using one-way analysis of variance (ANOVA). Homogeneity of variances was assessed using Levene’s test, with the Brown–Forsythe robust test prespecified in case of significant heteroscedasticity. Normality was assessed using the Shapiro–Wilk test. Given the unequal RT-qPCR group sizes, the robustness of the correlation findings was additionally examined using Spearman’s rank correlation. Pearson’s correlation coefficient (r) was used as the primary analysis to evaluate linear associations among the ΔCq values of NF-κB, GSK3β, and P21. All statistical tests were two-tailed, and statistical significance was established at *p* < 0.05. The intervertebral disc specimen was considered the experimental unit for all statistical analyses.

## 3. Results

### 3.1. Clinical Characteristics of the Study Population

Thirty-two patients undergoing surgical treatment for lumbar degenerative disc disease were prospectively enrolled in this study. Thirty patients with symptomatic lumbar degenerative disc disease comprised the study population included in the clinical and statistical analyses (Table 1), whereas two patients with Pfirrmann grade I intervertebral discs served exclusively as laboratory controls and were not included in the clinical or statistical analyses. A total of 36 surgically obtained intervertebral disc specimens were collected, with four patients contributing specimens from two lumbar levels. Because tissue availability differed after specimen processing, RT-qPCR analyses were performed on 34 specimens (6 mild and 28 severe degeneration), whereas oxidative stress biomarkers were evaluated in 32 specimens equally distributed between mild (n = 16) and severe (n = 16) degeneration.

### 3.2. Senescence-Related Gene Expression

**Figure 1:**
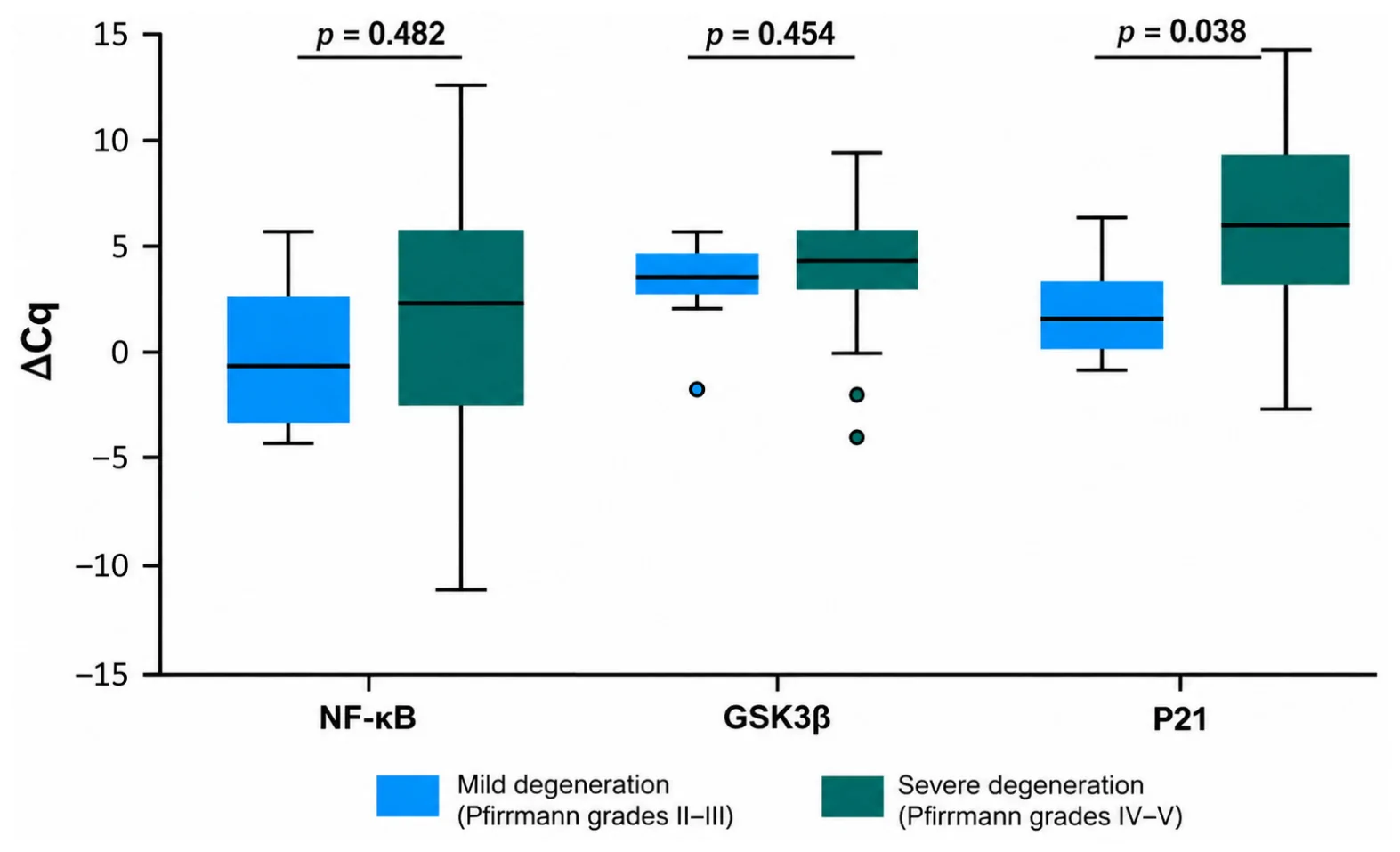
ΔCq values for NF-κB, GSK3β, and P21 in human intervertebral disc specimens with mild (Pfirrmann grades II–III) and severe (grades IV–V) degeneration. Boxes represent the median and interquartile range, whiskers indicate the minimum and maximum values, and statistical comparisons between groups were performed using one-way ANOVA. Circles indicate outliers (values more than 1.5 interquartile ranges beyond the box).

To investigate the relationship between disc degeneration severity and alterations in senescence-related molecular pathways, the expression of NF-κB, GSK3β, and P21 was quantified by RT-qPCR. ΔCq values were used for statistical comparisons between degeneration groups. P21 ΔCq values were significantly higher in severely degenerated discs (Pfirrmann grades IV–V) than in mildly degenerated discs (grades II–III) (6.24 ± 4.25 vs. 2.35 ± 2.20; *p* = 0.038), indicating lower relative P21 expression in the severe degeneration group. No statistically significant differences in ΔCq values were observed for NF-κB or GSK3β (Table 3, Figure 1).

#### Correlation Among Senescence-Related Genes

Pearson’s correlation analysis demonstrated significant positive correlations among all evaluated genes. The strongest correlation was observed between NF-κB and P21 (r = 0.790, *p* < 0.001), followed by GSK3β and P21 (r = 0.570, *p* < 0.001) and NF-κB and GSK3β (r = 0.550, *p* = 0.001). These findings indicate coordinated variation among the evaluated molecular pathways during lumbar disc degeneration (Table 4).

### 3.3. Biochemical Assessment of Oxidative Stress

#### Oxidative Stress Biomarkers

**Figure 2:**
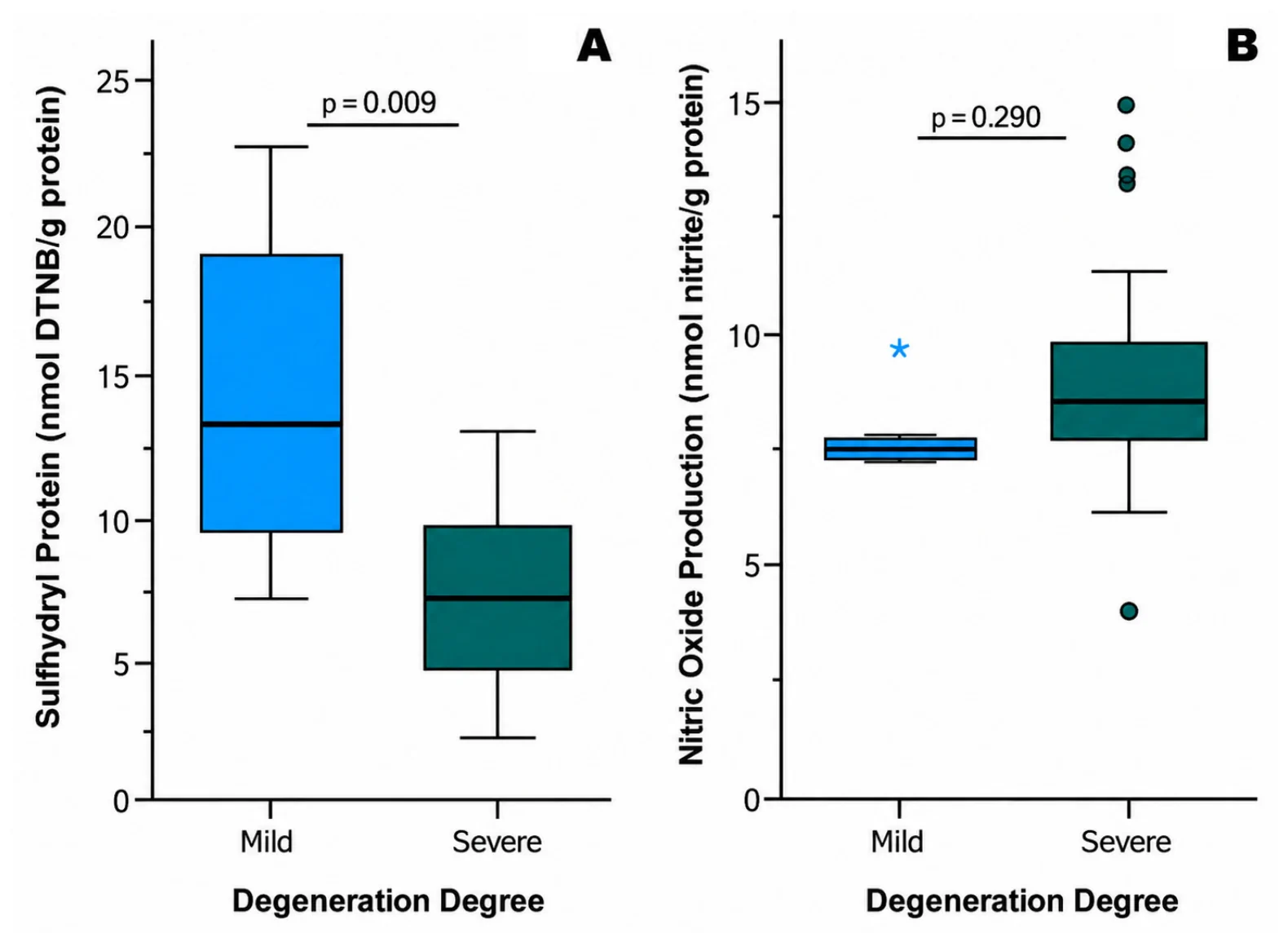
Oxidative stress biomarkers according to the severity of intervertebral disc degeneration. (**A**) Sulfhydryl protein levels (nmol DTNB/g protein) in mild and severe degeneration groups. (**B**) Nitric oxide production (nmol nitrite/g protein) in mild and severe degeneration groups. Statistical comparisons between groups were performed using one-way ANOVA. Circles represent outliers (values more than 1.5 interquartile ranges beyond the box), whereas asterisks indicate extreme values (more than 3 interquartile ranges beyond the box).

Oxidative stress was evaluated through quantification of sulfhydryl protein levels and nitric oxide production. Sulfhydryl protein levels were significantly higher in mildly degenerated discs than in severely degenerated discs (14.42 ± 6.37 vs. 7.52 ± 3.26; *p* = 0.009), indicating reduced antioxidant capacity in advanced stages of degeneration (Figure 2A). Nitric oxide production tended to be higher in severely degenerated discs than in mildly degenerated discs (9.29 ± 2.55 vs. 8.02 ± 0.92), although this difference did not reach statistical significance (*p* = 0.290) (Figure 2B).

## 4. Discussion

**Figure 3:**
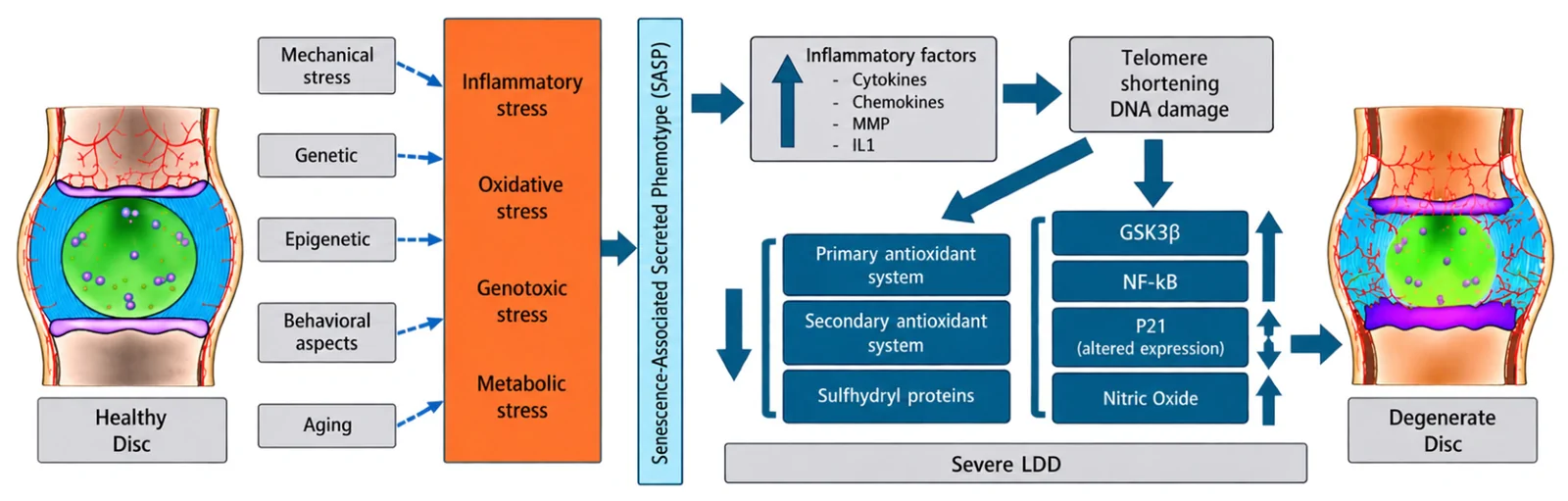
Conceptual overview of oxidative stress and senescence-related pathways potentially involved in lumbar degenerative disc disease. The diagram summarizes biological pathways discussed in the literature and provides a conceptual framework for the biomarkers evaluated in the present study; it does not imply increased expression of all depicted molecular markers. In the present study, higher P21 ΔCq values in severely degenerated discs indicated lower relative P21 expression. Arrows indicate the proposed direction of biological relationships between the depicted pathways and processes. The diagram is conceptual and does not represent experimentally demonstrated causal relationships in the present study.

The present study investigated the relationship between biochemical oxidative stress biomarkers and senescence-related gene expression in human lumbar intervertebral disc tissue at different stages of degeneration. To our knowledge, this is one of the few studies simultaneously evaluating oxidative stress biomarkers together with senescence-related molecular pathways in surgically obtained human disc tissue. Three principal findings emerged. First, advanced degeneration was associated with significantly reduced sulfhydryl protein levels, indicating impaired antioxidant capacity. Second, P21 ΔCq values were significantly higher in severely degenerated discs, indicating lower relative P21 expression and supporting an association with senescence-related molecular alterations. Third, significant positive correlations among the ΔCq values of NF-κB, GSK3β, and P21 suggest coordinated variation in these molecular pathways rather than independent molecular changes. Collectively, these findings reinforce the concept that oxidative stress and cellular senescence represent interconnected biological processes contributing to the progression of lumbar degenerative disc disease [15,16,18,19]. A conceptual overview of the oxidative stress and senescence-related pathways potentially involved in lumbar degenerative disc disease is presented in Figure 3.

Oxidative stress has emerged as one of the major mechanisms underlying intervertebral disc degeneration. Under physiological conditions, reactive oxygen species (ROS) participate in redox signaling and maintenance of tissue homeostasis. However, aging, mechanical overload, mitochondrial dysfunction, and chronic inflammation promote excessive ROS production, exceeding endogenous antioxidant capacity. This imbalance results in oxidative damage to DNA, proteins, and lipids, promotes extracellular matrix degradation, and amplifies inflammatory signaling through activation of transcription factors such as NF-κB, establishing a self-perpetuating degenerative microenvironment [8,10,11,15,16]. The present findings are consistent with this biological model, demonstrating simultaneous impairment of antioxidant defenses and senescence-related molecular alterations.

Among all evaluated genes, P21 was the only marker showing a statistically significant difference between mild and severe degeneration. P21 ΔCq values were significantly higher in severely degenerated discs, indicating lower relative P21 expression in this group. P21 is a key downstream effector of the p53 pathway and plays an important role in cell-cycle arrest and cellular senescence following cellular stress. Although previous studies have reported increased P21 expression in degenerated intervertebral discs, our findings indicate a different expression pattern in advanced degeneration. This discrepancy may reflect differences in disease stage, tissue composition, cellularity, or methodological approaches and highlights the complexity of senescence-associated signaling during disc degeneration [18,19,28,29,30,31,32,33,34].

Previous investigations have demonstrated NF-κB activation in degenerated intervertebral discs [35], alterations in mitochondrial gene expression in human annulus fibrosus cells [36], and the involvement of cellular senescence in intervertebral disc aging and degeneration [37]. Although NF-κB and GSK3β did not demonstrate statistically significant differences between degeneration stages, their ΔCq values tended to be higher in severely degenerated discs, corresponding to lower relative expression. More importantly, significant positive correlations among the ΔCq values of NF-κB, GSK3β, and P21 suggest coordinated variation in these molecular pathways rather than isolated changes. NF-κB is a central regulator of inflammatory signaling, whereas GSK3β participates in mitochondrial function, apoptosis, and oxidative stress responses [16,38,39,40,41,42,43,44,45,46,47].

The biochemical findings further reinforce the molecular observations. Sulfhydryl protein levels were significantly lower in severely degenerated discs, indicating progressive impairment of endogenous antioxidant defenses. Sulfhydryl-containing compounds represent one of the principal non-enzymatic antioxidant systems responsible for maintaining intracellular redox homeostasis [27,48]. Nitric oxide showed a tendency toward higher concentrations in severely degenerated discs, although statistical significance was not reached. Together, these findings support the concept that oxidative stress and cellular senescence constitute closely interconnected biological processes.

The present findings have important translational implications. Although this study was not designed to establish diagnostic or prognostic biomarkers, the simultaneous evaluation of biochemical oxidative stress markers and senescence-related gene expression provides a broader characterization of the biological events occurring during lumbar disc degeneration than studies based on a single methodological approach. These observations support future investigations evaluating antioxidant or senescence-modulating therapeutic strategies. Importantly, because molecular and biochemical analyses were performed on different subsets of specimens according to tissue availability, direct correlations between oxidative stress biomarkers and gene expression could not be established. Therefore, the present findings should be interpreted as complementary biochemical and molecular evidence rather than direct mechanistic interactions. Future studies evaluating both approaches in the same specimens are warranted.

The present study has several strengths. It is among the few investigations evaluating biochemical oxidative stress biomarkers together with senescence-related gene expression in surgically obtained human intervertebral disc tissue. The use of human specimens increases the translational relevance of the findings. Nevertheless, some limitations should be acknowledged, including the relatively small sample size, the cross-sectional design, and the limited panel of oxidative stress biomarkers evaluated. An additional limitation relates to the experimental unit. Although four patients contributed specimens from two lumbar levels, each intervertebral disc was analyzed as an independent anatomical specimen with its own radiological degeneration grade. Nevertheless, some degree of statistical dependence cannot be completely excluded and should be considered when interpreting the present findings. Larger prospective studies integrating biochemical, molecular, and proteomic approaches are warranted.

Overall, the present study provides additional evidence that oxidative stress and alterations in senescence-related molecular pathways are associated with lumbar degenerative disc disease. Reduced sulfhydryl protein levels, altered P21 expression, and significant correlations among NF-κB, GSK3β, and P21 support an association between impaired redox homeostasis and molecular changes accompanying disc degeneration.

## 5. Conclusions

This study provides evidence that advanced lumbar degenerative disc disease is associated with impaired antioxidant capacity and alterations in senescence-related molecular pathways in human intervertebral disc tissue. Severely degenerated discs exhibited significantly reduced sulfhydryl protein levels and significantly higher P21 ΔCq values, indicating lower relative P21 expression. Significant positive correlations among the ΔCq values of NF-κB, GSK3β, and P21 further suggest coordinated variation in these molecular pathways.

These findings support an association between impaired redox homeostasis and alterations in senescence-related molecular pathways during lumbar disc degeneration. The combined assessment of biochemical oxidative stress markers and gene expression provides complementary information on the biological changes accompanying disease severity.

Further studies involving larger cohorts, additional molecular markers, and longitudinal designs are warranted to clarify the biological significance of these findings and to investigate the potential role of oxidative stress and senescence-related pathways as therapeutic targets in lumbar degenerative disc disease.

## Figures and Tables

**Table 1:** Clinical characteristics of the 30 patients included in the clinical and statistical analyses.

Patient	Level	Pfirrmann	Age	Gender	Experiment
1	L4–L5	(Severe) IV	46	Female	RT-qPCR, OE
2	L4–L5 L5–S1	(Severe) IV (Severe) V	46	Male	OE OE
3	L4–L5	(Severe) V	68	Female	RT-qPCR, OE
4	L4–L5	(Severe) IV	46	Female	RT-qPCR, OE
5	L4–L5	(Severe) IV	49	Male	RT-qPCR, OE
6	L4–L5	(Severe) IV	39	Male	RT-qPCR, OE
7	L5–S1	(Severe) IV	35	Female	RT-qPCR, OE
8	L3–L4 L5–S1	(Severe) IV (Severe) V	72	Female	RT-qPCR, OE RT-qPCR, OE
9	L5–S1	(Severe) IV	51	Male	RT-qPCR, OE
10	L4–L5 L5–S1	(Severe) V (Severe) IV	57	Male	RT-qPCR, OE RT-qPCR, OE
11	L3–L4 L4–L5	(Severe) V (Severe) V	64	Female	RT-qPCR, OE RT-qPCR, OE
12	L5–S1	(Severe) V	39	Male	RT-qPCR, OE
13	L4–L5	(Severe) IV	66	Female	RT-qPCR, OE
14	L4–L5 L5–S1	(Severe) IV (Severe) IV	43	Female	RT-qPCR RT-qPCR
15	L4–L5	(Severe) IV	44	Male	RT-qPCR, OE
16	L5–S1	(Severe) V	38	Male	RT-qPCR, OE
17	L4–L5	(Severe) IV	48	Female	RT-qPCR, OE
18	L4–L5	(Severe) IV	46	Female	RT-qPCR, OE
19	L4–L5 L5–S1	(Severe) IV (Severe) V	59	Male	RT-qPCR, OE RT-qPCR, OE
20	L3–L4	(Severe) V	71	Male	RT-qPCR, OE
21	L5–S1	(Mild) III	29	Female	RT-qPCR, OE
22	L5–S1	(Mild) III	54	Female	RT-qPCR, OE
23	L5–S1	(Mild) II	27	Male	OE
24	L5–S1	(Mild) III	40	Female	RT-qPCR, OE
25	L3–L4	(Mild) III	53	Female	RT-qPCR, OE
26	L3–L4 L4–L5	(Mild) III (Mild) III	60	Male	RT-qPCR, OE RT-qPCR, OE
27	L5–S1	(Severe) IV	76	Male	RT-qPCR, OE
28	L5–S1	(Severe) IV	30	Female	RT-qPCR, OE
29	L4–L5	(Severe) IV	53	Female	RT-qPCR
30	L4–L5	(Severe) V	59	Male	RT-qPCR

**Table 2:** The genes and oligonucleotide sequences used in the research.

	Oligonucleotides Sequence	
Gene (GenBank)	Forward	Reverse
NF-kB (ID: 4790)	CAGCACTACTTCTTGACCACC	TCTGCTCCTGAGGATTGACGTC
GSK3β (ID: 173149)	CTGTTCCGAAGTTTAGCCTATAT	ACAAGAGGTTCTGCGGTTTA
P21/CDKN1A (ID: 1026)	CCCGTGAGCGATGGAACT	CCTGCCTCCTCCCAACTCAT
ACTB (ID: 60)	CTGTCGAGTCGCGTCCACC	TCCCACCATCACACCCTGG

**Table 3:** ΔCq values of NF-κB, GSK3β, and P21 according to the severity of intervertebral disc degeneration.

	Degree of Disc Degeneration		
	Mild (n = 6)	Severe (n = 28)	*p*
NF-kB	−0.32 ± 4.10	1.43 ± 5.66	0.482
GSK3β	3.02 ± 2.64	3.95 ± 2.75	0.454
P21	2.35 ± 2.20	6.24 ± 4.25	0.038 *

* Values are presented as mean ± standard deviation. ΔCq values were calculated relative to ACTB (β-actin). Statistical comparisons between mild and severe degeneration groups were performed using one-way ANOVA. *p* < 0.05.

**Table 4:** Correlation of genetic expression between NF-kB, GSK3β and P21 (Pearson, *p* ≤ 0.001 **).

		NF-kB	GSK3β	P21
**NF-kB**	r	1	0.550 **	0.790 **
	*p*-value		0.001	<0.001
**GSK3β**	r	0.550 **	1	0.570 **
	*p*-value	0.001		<0.001
**P21**	r	0.790 **	0.570 **	1
	*p*-value	<0.001	<0.001	

## Data Availability

The data presented in this study are available on request from the corresponding author.
